# Mitochondrial transplantation in lung diseases: From mechanisms to application prospects

**DOI:** 10.1016/j.gendis.2025.101856

**Published:** 2025-09-18

**Authors:** Haoneng Wu, Qiuran Zhao, Ying Zhao, Jinguang Bai, Junxi Pan, Songling Huang

**Affiliations:** a Yunnan Key Laboratory of Laboratory Medicine, Kunming, Yunnan 650032, China; b Yunnan Province Clinical Research Center for Laboratory Medicine, Kunming, Yunnan 650032, China; c Department of Clinical Laboratory, The First Affiliated Hospital of Kunming Medical University, Kunming, Yunnan 650032, China; d The First People’s Hospital of Lushui City, Lushui, Yunnan 673200, China

**Keywords:** Lung disease, Mitochondrial dysfunction, Mitochondrial transplantation, Oxidative stress, Respiratory system

## Abstract

Mitochondria are double-membrane organelles in eukaryotic cells, which play an important role in energy metabolism, cell cycle and apoptosis. Therefore, mitochondrial abnormalities can affect various physiological and pathological processes. Extensive research over a long period of time has shown that mitochondrial dysfunction is considered a hallmark of several diseases, including cardiovascular diseases, neurodegenerative diseases, respiratory diseases, and even cancer. Mitochondrial transplantation has emerged in recent years as a novel approach for treating mitochondria-related diseases. This therapy involves transferring viable, functionally intact mitochondria into cells or tissues, either directly or indirectly, to replace dysfunctional mitochondria and restore mitochondrial function, thereby achieving therapeutic goals. Research has indicated that mitochondrial transplantation can alleviate the progression of lung diseases and improve disease outcomes. In this review, we explore the mechanisms underlying mitochondrial dysfunction in lung disease and the potential application of mitochondrial transplantation in the treatment of lung disease.

## Introduction

Due to prolonged exposure to gas exchange with the external environment, the respiratory system is particularly susceptible to damage mediated by reactive species (RS) such as reactive oxygen species (ROS) and reactive nitrogen species (RNS). To prevent or mitigate this damage, the respiratory system typically possesses corresponding antioxidant substances to maintain an “oxidative-antioxidative” balance.^1^ However, this balance has been disrupted in various lung diseases, including pulmonary fibrosis,^2^ chronic obstructive pulmonary disease (COPD),^3^ and pulmonary arterial hypertension (PAH),^4^ leading to oxidative stress in the respiratory system. The dysregulation of the “oxidative-antioxidative” balance is mediated by an increase in oxidative levels and a decrease in antioxidant capacity. For instance, in the lungs of COPD patients, increased oxidative stress and reduced antioxidant capacity have been observed, along with the inactivation of antioxidant enzymes and various transcription factors regulating antioxidant genes.^3^ An important contributor to oxidative stress is the elevated level of ROS.

Reactive oxygen species (ROS) are byproducts of the mitochondrial electron transport chain (ETC), primarily consisting of superoxide anions, hydrogen peroxide, and hydroxyl radicals.^5^, ^6^, ^7^ Previously, it was believed that ROS were associated with the onset of various diseases and poor prognosis; however, it is now evident that ROS play a crucial role in cellular signal transduction, regulating numerous physiological processes, such as cell proliferation, differentiation, aging, and apoptosis.^8^ As significant oxidants within the body, an imbalance between the excessive production of ROS and their neutralization can induce oxidative stress, leading to various diseases.^9^ Due to prolonged exposure to endogenously generated ROS from mitochondrial respiration, the lungs are susceptible to ROS-mediated oxidative damage.^3^ Therefore, correcting mitochondrial damage in the lungs may be a potential therapeutic strategy for lung diseases.

Mitochondria are the primary sites of ROS production within cells. Under physiological conditions, 0.2%–2% of electrons in the ETC do not undergo normal transfer; instead, they leak from the ETC and react with oxygen to form superoxide and hydrogen peroxide.^8^^,^^10^ Generally, physiological levels of ROS do not cause significant negative effects on the body; however, elevated levels of ROS can mediate oxidative damage to tissues and cells, leading to mitochondrial dysfunction. Mitochondrial dysfunction represents a central feature of diverse pathological conditions, including neurodegenerative diseases (such as Alzheimer’s disease),^11^ cardiovascular diseases (such as heart failure),^12^ and chronic lung diseases (such as chronic obstructive pulmonary disease, COPD).^13^ Furthermore, a prominent characteristic of mitochondrial dysfunction is damage to mtDNA, encompassing impaired mtDNA expression, increased mtDNA mutations, and reduced mtDNA copy number.^14^ These alterations further lead to the loss of the mitochondrial membrane potential and increased outer membrane permeability, resulting in the release of pro-apoptotic factors that drive disease development.^15^ Interestingly, beyond mtDNA, microRNAs (miRNAs) encoded within the mitochondrial genome are also implicated in the regulation of mitochondrial functions, including oxidative phosphorylation and ATP synthesis.^16^ Therefore, the role of microRNAs in mitochondrial transplantation deserve further study in the future.

For mitochondrial dysfunction in pulmonary diseases, mitochondrial transplantation may be a viable treatment. Mitochondrial transplantation is the transfer of active mitochondria into damaged cells, either directly or indirectly, with the aim of restoring mitochondria that function abnormally in mitochondria-deficient cells.^17^ As early as the last century, isolated mitochondria have been transferred into another type of cell.^18^ In recent years, mitochondrial transplantation techniques have advanced rapidly and have been successfully applied with good efficacy in various disease models, including ischemia-reperfusion injury,^19^ Parkinson’s disease (PD),^20^ and breast cancer.^21^ The potential efficacy of mitochondrial transplantation has likewise been observed in lung diseases. Research by Sun et al indicated that, regardless of whether used in conjunction with melatonin, mitochondrial therapy significantly improves lung and circulatory function in rat models of acute respiratory distress syndrome (ARDS), which is achieved mainly through the inhibition of inflammation, oxidative stress, DNA damage and apoptosis.^22^ Therefore, it is not difficult to speculate that active mitochondria, after transplantation into cells, can replace mitochondria with abnormal functions, restore the mtDNA damage repair system, reduce ROS-mediated oxidative stress, and perform normal mitochondrial function.

## Mitochondrial dysfunction in lung diseases

### Susceptibility to pulmonary oxidative stress

As the organ with the largest surface area in the human body, the lung is constantly exposed to exogenous ROS stimulation, such as CO_2_, SO_2_, CO, atmospheric particles and cigarette smoke, due to the need for gas exchange with the external environment, which results in the need for a powerful stress response pathway in the lung to avoid cumulative damage.^15^^,^^23^ In addition to exogenous ROS, endogenous ROS are mainly synthesized in mitochondria, which are mostly catalyzed by NADPH oxidase (NOX), myeloperoxidase (MPO), nitric oxide synthase and xanthine oxidase expressed by macrophages and neutrophils and are used to resist the invasion of bacteria, fungi, viruses and others.^24^ Since ROS modification of nearby biomolecules is non-selective, the body requires antioxidant defense systems to protect against oxidative stress caused by ROS, including superoxide dismutase, catalase, peroxidase, glutathione systems, and vitamins or amino acids that have direct antioxidant effects or act as precursors or cofactors for antioxidant enzymes.^25^

In addition to the antioxidant defense system, autophagy also plays an important role in maintaining redox homeostasis. When the levels of ROS are too high or the oxidative-antioxidant balance is dysregulated, the oxidative stress triggered leads to oxidative damage to biomolecules such as proteins, lipids, and nucleic acids, which affects normal cellular activities and functions.^26^ Moreover, oxidative stress can also induce the activation of the autophagy pathway to scavenge damaged biomolecules and maintain cellular homeostasis; therefore, autophagy is also considered as a secondary defense against oxidative stress.^27^ As the main site of ROS production, mitochondria play a crucial role in autophagy. Prolonged impairment of mitochondrial function triggers the generation of large amounts of ROS, which induces the activation of the mitochondrial autophagy pathway and prompts mitochondrial self-cleaning to prevent the generation of mitochondrial ROS (mtROS) and protect the cells from oxidative stress.^28^ However, when autophagy is disrupted, ROS-producing mitochondria continue to accumulate, resulting in further excessive release of ROS and exacerbating cellular oxidative damage.^29^ Recent studies have shown that mitochondrial dysfunction and dysregulation of redox homeostasis have been observed in a variety of lung diseases, including COPD, idiopathic pulmonary fibrosis (IPF), asthma, and PAH, which not only causes oxidative stress in lung tissues, but also leads to alterations in the mitochondrial autophagy phenotype.^27^^,^^30^^,^^31^ For instance, one study noted that exposure to an air pollutant, tetrabromobisphenol A (TBBPA), induced an overproduction of ROS in the lungs of mice, leading to activation of the NF-κB/TNF-α inflammatory pathway and impaired tissue architecture.^32^ Additionally, this study indicated that elevated ROS levels can reduce the activity of the antioxidant defense system, inhibit autophagy, and promote apoptosis,^32^ suggesting that the dysregulation of redox homeostasis induced by ROS affects the progression of lung diseases from multiple aspects.

### MtDNA damage and escape

Mitochondrial DNA (mtDNA) is considered to be a residual circular DNA molecule within the original bacteria, approximately 16.5 kb in mammals, and is responsible for encoding 13 essential proteins of the oxidative phosphorylation (OXPHOS) system.^33^ As mentioned earlier, mtDNA is highly susceptible to oxidative damage and exhibits a mutation rate that is significantly higher than that of nuclear DNA (nDNA). Moreover, mitochondria play crucial roles in regulating various signaling pathways, energy metabolism, cell differentiation, proliferation, and death,^34^ which makes mitochondrial dysfunction and mtDNA mutations widely present in disease processes. In addition to the coding region, the D-loop region of mtDNA serves as a non-coding region that contains the replication origin and transcription promoters, which implies that the D-loop region is a regulatory region for mtDNA replication and transcription.^35^ Compared to that of nuclear DNA, the mutation rate of mtDNA is 10–20 times higher, with the mutation rate in the D-loop region being an astonishing 100 to 200 times greater.^36^ What is known at present is that the high mutation rate of mtDNA is closely associated with ROS-mediated oxidative damage, which can be explained by several reasons, including the fact that mtDNA is located in the mitochondrial matrix, near the ROS generation site of the ETC, and is more susceptible to ROS-mediated oxidative damage.^37^ In addition, mtDNA is barely “nucleoid” and lacks protective histones, which also makes mtDNA more sensitive to oxidative damage.^38^ It is noteworthy that mammals have evolved a variety of mechanisms to repair DNA damage induced by various factors in order to ensure genomic stability.^39^ However, compared to that of nuclear DNA, the damage repair capacity of mtDNA is limited. A study on emphysema found that type II alveolar epithelial cells in emphysema patients exhibited significantly increased mitochondrial superoxide production, extensive mtDNA damage, and abnormalities in the DNA repair system.^40^ These pathological alterations may be associated with excessive ROS production. Base excision repair (BER) is considered the primary repair pathway for the mitochondrial genome, responsible for correcting forms of oxidative, deamination, alkylation, and abasic single-base damage.^41^^,^^42^ In addition to BER, other mtDNA repair pathways, such as mismatch repair (MMR), homologous recombination (HR), and non-homologous end joining (NHEJ), have also been suggested.^38^ However, there is also a viewpoint that although the proteins involved in these mechanisms are localized in mitochondria, substantial evidence is still lacking.^43^ Therefore, it is controversial whether these repair pathways are relevant to the maintenance of mtDNA.

Generally, mtDNA is present in the mitochondrial matrix; however, under certain stress conditions, mtDNA may escape into the cytoplasm or extracellular space, triggering inflammatory responses, but the mechanism of mtDNA escape has not been fully elucidated.^44^ McArthur et al found that mtDNA escape is mediated by BAK/BAX proteins.^45^ Following mitochondrial damage, BAK and BAX are activated, inducing outer mitochondrial membrane permeability, which leads to the release of cytochrome c and the activation of the apoptosis protein caspase, ultimately resulting in the mitochondrial network breakdown and the appearance of large BAK/BAX pores in the outer membrane. These BAK/BAX macropores allow the inner mitochondrial membrane to herniate into the cytosol, carrying the mitochondrial genome and triggering the escape of mtDNA. In addition to BAK/BAX pores, mtDNA escape mechanisms include the coordinated action of voltage-dependent anion channels (VDACs) and the mitochondrial permeability transition pore (mPTP) in the outer mitochondrial membrane (OMM), gasdermin pore, alteration of mitochondrial autophagy and lysosomes, and the active release of mtDNA via extracellular vesicles.^33^ Due to space limitations, this paper will not elaborate further; please refer to the reference 33 for more details. The escape of mtDNA is not only related to the permeability of mitochondria but also closely associated with the nature of mtDNA itself. Reports have indicated that ROS and other oxidants can induce mtDNA oxidation and that oxidized mtDNA (ox-mtDNA) is either repaired by BER or cleaved by the endonuclease FEN1 into fragments of 500–650 bp; these ox-mtDNA fragments can escape from the mitochondria through mPTP and VDAC-dependent channels, bind to the inflammatory body NLRP3 and activate NLRP3, leading to the secretion of pro-inflammatory cytokines, such as IL-1β and IL-18, thereby initiating an inflammatory response.^46^, ^47^, ^48^ Additionally, mtDNA in cytoplasm can activate the cGAS-STING pathway and induce the release of type I interferons (IFNs), that is, mtDNA is recognized by the DNA sensing ring cyclic guanosine monophosphate (GMP)-adenosine monophosphate (AMP) (cGAMP) cyclic GMP-AMP synthase (cGAS), which then activates STING and downstream signaling pathways.^49^ Since cGAS can sense any form of double-stranded DNA, mtDNA, whether oxidized or not, can activate the cGAS-STING pathway, with ox-mtDNA seemingly inducing a stronger immune stimulation response.^48^^,^^50^ Giordano et al demonstrated that cigarette smoke can induce depolarization of the mitochondrial membrane in bronchial epithelial cells, increase oxidative stress and mtDNA leakage.^51^ They also observed free mtDNA in the plasma of smokers with COPD, which led to the up-regulation of NLRP3 and cGAS-mediated pro-inflammatory cytokines.^51^

**Figure 1:**
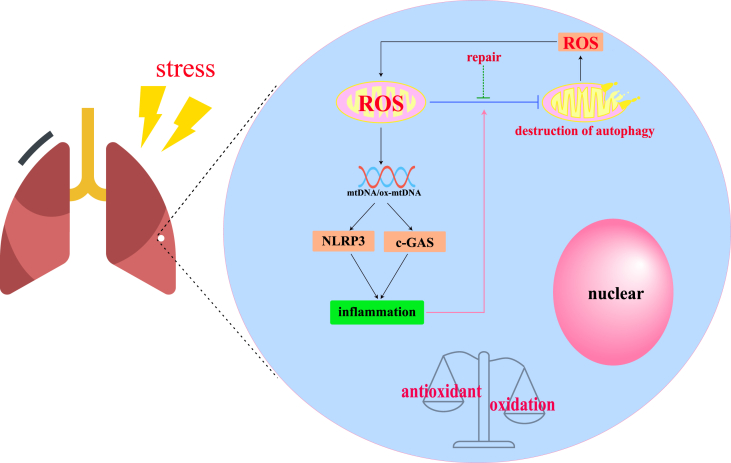
Mitochondria of lung cells under stress. Under stress, the “oxidative-antioxidative” balance of the lung is disordered, and the level of lung mitochondrial ROS increases, triggering an inflammatory response. After the repair system and mitochondrial autophagy are disrupted, the mitochondria release a large amount of ROS, which further triggers an inflammatory response, causing a vicious circle.

Based on the above discussion, we hypothesize that despite various antioxidant defense mechanisms, lung tissue is still subject to oxidative stress from environmental factors. When the redox system becomes unbalanced, mitochondrial function is impaired and this leads to an elevation in ROS levels, which mediates oxidative stress in lung tissue. As a result, mitochondrial DNA (mtDNA) is oxidized and may escape from the mitochondria. This triggers the activation of the NLRP3 inflammasome and the cGAS pathway, which in turn initiates inflammatory responses. If the mtDNA repair system fails to correct the damage to mtDNA, it can eventually trigger mitochondrial autophagy or induce apoptosis. When autophagic function is disrupted, excessive ROS are released, further exacerbating this process and promoting a vicious cycle of oxidative damage (Fig. 1).

### Feasibility of mitochondrial transplantation

Mitochondrial dysfunction can cause a wide range of diseases, and such diseases caused by mitochondrial dysfunction are often defined as mitochondrial diseases. Due to the heterogeneity of mitochondrial diseases, the diagnosis, management, and development of effective therapies for these diseases are extremely limited.^52^ At present, clinical approaches are based on pharmacological treatments, which are mostly aimed at supportive and symptomatic treatments that do not significantly alter the course of the disease.^53^^,^^54^ Mitochondrial transplantation, involving the transfer of normal mitochondria into cells to replace defective ones, is considered one of the most therapeutically promising approaches,.^55^ Although mitochondrial transplantation has not been reported to be a complete cure for mitochondrial diseases, the therapy has shown significant results in restoring the function of defective mitochondrial cells.^55^

### Source of mitochondria for transplantation

Generally speaking, there are no special requirements for the source of mitochondria used for transplantation, and almost any normal tissue or cell far from the lesion area can be used for mitochondrial isolation.^17^ However, mitochondria from different tissues and cells vary in terms of number, size, shape, distribution, and feature.^56^ Therefore, the source of donor mitochondria still requires comprehensive consideration from multiple aspects. In animal models, various tissues and cells have been used as mitochondrial donors, such as liver^57^ and skeletal muscle,^58^ which are notable for their abundance of mitochondria. While other tissues or cells have also been used as mitochondrial donors, tissues or cells with a high mitochondrial content are likely a more suitable choice, provided that other conditions remain consistent. A study has shown that mitochondria isolated from skeletal muscle cells, hepatocytes, and mesenchymal stem cells were treated with mitochondrial transplants in septic rats and that mitochondrial transplants from each cell type improved mitochondrial function; however, mitochondria from skeletal muscle cells exhibited more robust oxidative phosphorylation and higher rat survival rates, which implies that the effect of mitochondrial transplants on sepsis in rats depends on the cell type of mitochondrial origin.^59^ Additionally, given that tissues such as skeletal muscle and liver are more difficult to obtain clinically, platelet-derived mitochondrial transplantation has been proposed and studied, and the results showed that platelet-derived mitochondrial transplantation successfully improved neuronal mitochondrial function and cognitive deficits in mice.^60^ These findings may provide new insights for future mitochondrial donor sources in transplantation.

### Mechanisms of mitochondrial intercellular transfer

**Figure 2:**
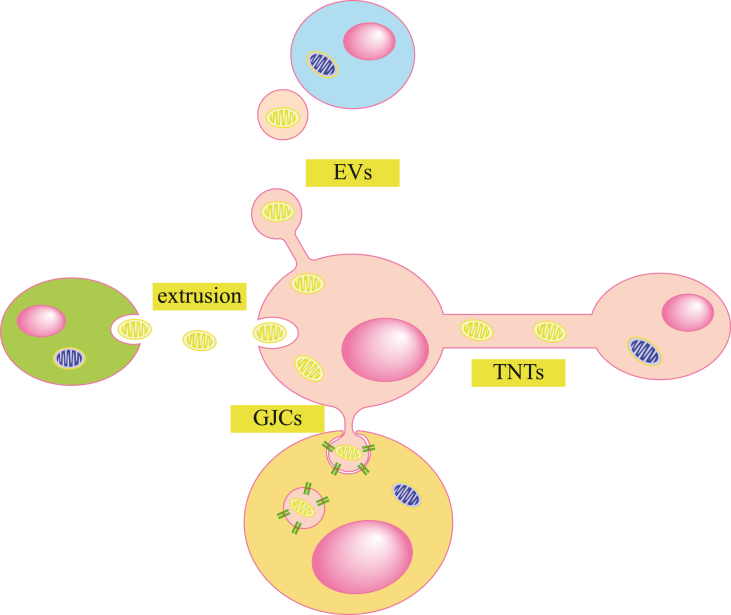
Mechanisms of mitochondrial intercellular transfer. These include tunneling nanotubes (TNTs), extracellular vesicles (EVs), extrusions, and gap junction channels (GJCs). The mitochondria in yellow are donor cell mitochondria, and those in blue are the recipient cell’s own mitochondria.

Mitochondria are highly dynamic organelles that constantly undergo a balance between fusion and fission, processes crucial for maintaining mitochondrial function and cellular homeostasis.^61^ In addition, mitochondria can act as a mediator of intercellular signaling and cellular communication through intercellular mitochondrial transfer. It has been shown that mitochondria can be transferred between cells via tunneling nanotubes (TNTs), extracellular vesicles (EVs), gap junction channels (GJCs), and mitochondrial extrusion.^62^^,^^63^ This intercellular mitochondrial transfer involves the transfer of mitochondrial genes or entire mitochondria into recipient cells, which may lead to changes of the host’s bioenergetic state, cell differentiation, cell survival, inflammatory responses, and drug resistance.^64^ Therefore, it is not difficult to understand the important role of intercellular mitochondrial transfer in regulating the physiological functions of various organ systems under both healthy and diseased conditions, including the regulation of cellular metabolism, the immune system, tissue homeostasis and cancer.^65^ In this section, we will briefly review the various mechanisms of mitochondrial transfer (Fig. 2).

### Tunneling nanotubes (TNTs)

In 2004, Rustom et al described a highly complex nanotube structure formed between cells that facilitated the selective transfer of membrane vesicles and organelles.^66^ Over the following decade, TNTs were shown to transfer cargos such as ions, pathogens, genetic material and proteins.^67^ Intercellular connectivity through the construction of TNTs has been observed in a variety of experimental models, including not only TNT construction between cells of the same type,^68^ but also between different cell types^69^ and even between tumor and non-tumor cells,^70^ which seems to imply that the establishment of inter-cellular TNTs is not limited by cell type. Although it is generally accepted that TNTs are the main cellular structure mediating mitochondrial intercellular transfer, the factors that stimulate TNT formation remain to be elucidated.^71^ Some scholars argue that TNTs play significant roles in both normal physiological functions and disease progression, which suggests that physiological processes or disease factors can trigger the formation of TNTs.^72^ A recent study also showed that microglia use TNTs to connect with neurons under physiological or pathological conditions and exert neuroprotective functions by transferring healthy mitochondria to reduce oxidative stress in neurons.^73^

### Extracellular vesicles (EVs)

Extracellular vesicles are small lipid-membrane vesicles secreted into the extracellular space and can be secreted by almost all cell types.^74^ Based on their size and the pathways through which they are synthesized, EVs can be classified as exosomes, ectosomes (also called microvesicles), and apoptotic bodies.^75^ EVs can transport cargoes such as nucleic acids, proteins, and lipids between cells, and the concentration of these cargos in EVs is higher than that in the environment; therefore, EVs are thought to be important in cellular communication.^75^^,^^76^ Research has found that brain endothelial cells (BECs) can transfer functionally normal mitochondria via microvesicles (the larger EV fraction) into oxygen-glucose deprived BECs (an *in vitro* model of cerebral ischemia) and increase their adenosine triphosphate (ATP) levels by 100–200 fold, suggesting that mitochondria transported via vesicles can play a protective role against ischemic.^77^ In addition, EVs have also been shown to play an important role in mitochondrial quality control (MQC). When mitochondrial dysfunction occurs, damaged mitochondria release mitochondrial components (such as mtDNA and mitochondrial proteins) into the cytoplasmic or extracellular environment, which can be recognized by immune cells as damage-associated molecular patterns (DAMPs) and mediate inflammatory responses.^78^ Consequently, in such situation, cells initiate the mitochondrial autophagy pathway to isolate defective mitochondria and deliver them to lysosomes for degradation.^79^ Interestingly, when lysosomes also show impaired function, there is a replacement pathway for mitochondrial elimination unrelated to autophagy, whereby damaged mitochondria are secreted through large EVs and subsequently captured by macrophages, with this pathway not causing the onset of an inflammatory response.^80^

### Mitochondrial extrusion

The earliest example of mitochondrial extrusion is the process of erythropoiesis described by Simpson et al. It was noted that the initial stage of mitochondrial extrusion is that vesicles from enucleated reticulocytes are attracted to the mitochondria to form large vacuoles encasing the mitochondria, which then fuses with the plasma membrane, releasing the mitochondria to the outside of the cell.^81^ In addition to physiological mitochondrial extrusion, mitochondria can also be extruded through autophagy. It has been observed that autophagosomes with mitochondria do not fuse with lysosomes but directly with the plasma membrane, releasing mitochondria in a process similar to secretory autophagy.^82^ In addition, Nakajima et al demonstrated another mitochondrial extrusion that does not depend on the autophagy pathway, showing that cytoplasmic vacuoles originating from the plasma membrane engulfed fragmented mitochondria during acute tumor necrosis factor α (TNF-α)-induced caspase-dependent cell death and extruded the mitochondria and their components into the extracellular space by exocytotic manner.^83^ Notably, intact actin and tubulin cytoskeleton are required for mitochondrial extrusion.^83^ After mitochondria extrusion, free mitochondria are transferred into target cells under certain specific conditions (such as co-incubation), a process also known as mitochondrial internalization,^84^ which will be discussed later. Indeed, it has been noted that free mitochondria or their components can be extruded or internalized in the absence of a membrane carrier.^85^ Regrettably, we have not yet found relevant evidence to prove the accuracy of his claim. However, it is certain that the process of mitochondrial extrusion into the extracellular space and subsequent uptake by target cells does not require carrier-mediated transport and can be achieved simply through co-incubation.

### Gap junction channels (GJCs)

A gap junction channel is composed of two hemichannels (also known as connexons), each of which is in turn a complex formed by the radial interaction of six proteins known as connexins (Cxs).^86^ In human beings, 21 Cx proteins have been identified, among which Cx43 is the most abundant and widely expressed in various organs and tissues.^87^ As direct intercellular communication channels, GJCs allow the passage of small molecules (those with a molecular weight less than 10^3^ kDa), such as inorganic salts, glucose, prostaglandins, and secondary messengers.^88^ Li et al has shown that bone marrow mesenchymal stem cells (BMSCs) transfer mitochondria to injured motor neurons via gap junctions, a process potentially involving heterotypic GJCs formed by Cx43 and Cx32.^89^ However, it is worth noting that mitochondria are much larger than the aperture size of GJCs, and the classical theory of GJCs cannot explain how they mediate mitochondrial transfer. One hypothesis suggests that donor cells form gap junctions with recipient cells, after which the donor cells release mitochondria in vesicles, then the recipient cells capture the vesicles by endocytosis to complete the mitochondrial transfer.^90^ However, it is not clear how internalized gap junctions release mitochondria, possibly through the transfer of contents generated by mitochondria into the cytoplasm of recipient cells via GJCs. Research by Islam et al also indicates that BMSCs form Cx43-containing GJC (Cx43-GJC) with alveolar epithelial cells, releasing mitochondria-containing microvesicles phagocytosed by the epithelial cells,^91^ which may imply that the GJCs do not mediate the transfer of mitochondria directly, but participate indirectly in other ways.

### Artificial mitochondrial isolation methods

Mitochondrial components, such as mtDNA and mitochondrial proteins, have been shown to activate the NLRP3 inflammasome, thereby inducing immune responses.^78^ Therefore, to prevent such adverse events, it is essential to isolate intact, active mitochondria from cells for successful mitochondrial transplantation. Common artificial mitochondrial isolation methods include differential centrifugation and density gradient centrifugation.^92^, ^93^, ^94^, ^95^ However, these isolation procedures often take approximately 1–2 h, and the entire isolation process needs to be performed at a low temperature of 4 °C, which limits the clinical applicability of these methods.^96^^,^^97^

Acín-Pérez et al summarized mitochondrial isolation methods for mouse liver, heart, kidney, skeletal muscle, brain, and brown and white adipose tissues, which varied considerably from one tissue to another in terms of the composition of the isolation buffer used, the method of tissue fragmentation, the speed of centrifugation, and sometimes the need for a specific step to improve quality and yield; however, these methods relied on the fundamental principle of differential centrifugation.^94^ Differential centrifugation separates mitochondria based on the sedimentation rate of the particles, allowing for efficient and rapid mitochondrial isolation. For detailed procedures, refer to Acín-Pérez et al.^94^ In addition to tissue mitochondrial extraction, Wettmarshausen et al also proposed a differential centrifugation-based method for isolating mitochondria from cells, which is similar to the mitochondrial extraction method used for tissues.^95^

Density gradient centrifugation is mostly used for mitochondrial purification and contaminant removal.^92^^,^^95^ Generally, non-mitochondrial components such as cell fragments cannot be completely removed by differential centrifugation. In order to improve the purity of mitochondria, differential centrifugation can be combined with a discontinuous Percoll density gradient to purify mitochondria.^95^ However, this option often comes at the cost of function and yield of extracted mitochondria; therefore, the method of mitochondrial extraction needs to be chosen according to the purpose of the experiment and the demand for mitochondria.^94^

To address the problem of long mitochondrial extraction times, a method called Differential Filtration has been proposed,^98^ which uses the tissue dissociator’s standardized homogenization cycle instead of manual homogenization to achieve uniform and consistent homogenization that is difficult to achieve with manual homogenization. In addition, the method uses a series of graded filter filtrations instead of the time-consuming and repetitive centrifugation steps of differential centrifugation, which is why this method can achieve the separation of active mitochondria within 30 min.

## Methods of mitochondrial delivery

### Mechanisms of mitochondrial internalization

Mitochondrial internalization refers to the process of transferring isolated mitochondria into target cells. Currently, the mechanisms of mitochondrial internalization remain to be explored. A study on mitochondrial transplantation in the ischemic myocardium by Pacak et al demonstrated that mitochondria were internalized into cardiomyocytes in a time-dependent manner via co-incubation, and that mitochondrial internalization was inhibited and the ATP content was reduced after the administration of an actin blocker, suggesting that mitochondrial internalization was mediated by actin-dependent endocytosis.^99^ Similarly, a study by Sun et al also indicated that actin-dependent endocytosis is involved in the internalization of exogenous mitochondria into glioma cells and this process is mediated by the NAD^+^-CD38-cADPR-Ca^2+^ signaling pathway.^100^ Interestingly, research by Kitani et al showed that the uptake of mitochondria by cardiomyocytes involves macropinocytosis,^84^ which contradicts the findings of Pacak et al, who suggested that while macropinocytosis plays a role in mitochondrial internalization, it does not appear to be involved in the internalization of mitochondria into cardiomyocytes.^99^

### Methods of mitochondrial transplantation *in vivo* and *in vitro*

In 1982, Clark and colleagues isolated mitochondria from chloramphenicol (CAP) and efrapeptin (EF)-resistant cells and transferred these mitochondria into CAP and EF-sensitive cells via co-incubation, which resulted in mitochondrial receptor cells with stable resistance to antibiotics.^18^ In subsequent decades, several mitochondrial transplantation methods have been applied. In addition to co-incubation, mitochondrial transfer methods *in vitro* also include direct microinjection,^101^ mitoception,^102^ cell-penetrating peptide,^103^ magnetomitotransfer,^104^ photothermal nanoblade^105^ and other methods (Table 1). Furthermore, *in vivo* mitochondrial transplantation methods often involve directly injecting isolated mitochondria into the affected area or into blood vessels^19^^,^^106^^,^^107^ (Table 1).

**Table 1:** Mitochondrial transplantation methods *in vivo* and *in vitro*.

Methods	Experimental model	Source of mitochondria	Target cells/tissues	Type of Study (*in vivo*/*in vitro*)	Brief introduction of experimental process	Results	Reference
Co-incubation		Cells resistant to chloramphenicol and efrapeptin	Cells sensitive to chloramphenicol and efrapeptin	*In vitro*	Co-incubation of purified mitochondria with target cells	Mitochondrial receptor cells were stably resistant to chloramphenicol and efrapeptin.	18
Co-incubation		Normal human astrocytes (HA)	Human glioma cells (U87)	*In vitro*	Co-incubation of purified mitochondria with starved U87 cells	Mitochondria were transferred to U87 cells, where aerobic respiration was enhanced, glycolysis was attenuated, the mitochondrial apoptotic pathway was reactivated, and malignant proliferation was inhibited.	100
Direct microinjection		Mouse liver tissues	Mouse zygotes	*In vitro*	Injection of mouse liver mitochondria into mouse zygotes using microinjection tools	Zygotes with mitochondrial transplants developed better than those without transplants.	108
Mitoception		Human mesenchymal stem cells (MSCs)	MDA-MB-231 cells	*In vitro*	The mitochondrial suspension was slowly added to the MDA-MB-231 cell culture medium and centrifuged at 1500 g for 15 min at 4 °C.	Mitochondrial transplantation increased the endogenous mitochondrial pool and enhanced energy metabolism and functional properties of MDA-MB-231 cells	102
Cell-penetrating peptide		Mouse liver	Stroke-like episodes (MELAS) hybrid cells containing the A3243G mutation	*In vitro*	Mitochondrial transplantation mediated by the cell-penetrating peptide Pep-1	Mitochondrial transfer restored respiratory function and increased biosynthesis in hybrid cells.	103
Magneto-mitotransfer		Human fetal lung fibroblast cell line MRC-5	Human fetal lung fibroblast cell line MRC-5	*In vitro*	Anti-TOM22 magnetic bead-labeled mitochondria were added to recipient cells and placed on a magnetic plate, using a magnetic field to pull the mitochondria into the cells.	Magnetomitotransfer efficiency outranged the efficiency of co-incubation, and enhanced respiration of recipient cells.	104
Photothermal nanoblade		Human breast carcinoma cells MDA-MB-453	Human osteosarcoma cells 143BTK	*In vitro*	Using a nanoblade to form a channel a few micrometers long on the surface of the recipient cell membrane, mitochondria packed inside the nanoblade were injected into the cell.	Mitochondrial transfer by photothermal nanoblade rescued the pyrimidine auxotroph phenotype and respiration of cells that lack mtDNA.	105
Direct injection into the affected area	Mice lower limb ischemia-reperfusion injury (IRI) model	Human umbilical cord mesenchymal stem cells (hMSCs)	Lower limb gastrocnemius muscle of C57BL/6 male mice	*In vivo*	Isolated mitochondria were injected into the gastrocnemius muscle 15 min after restoration of perfused mice lower limb.	Mitochondrial transplantation supplements the original damaged mitochondrial function of skeletal muscle, reduces apoptosis, restores muscle tissue, and promotes recovery of motor function *in vivo*.	106
Intravenous injection	Parkinson’s disease (PD) model	Human hepatoma cell HEPG2	Multiple tissues including brain in C57BL/6J male mice	*In vivo*	The mitochondrial solution was injected slowly via tail veins.	Intravenously injected mitochondria were widely distributed in brain, liver, kidney and other tissues, increasing the activity of the electronic respiratory chain, decreasing the level of reactive oxygen species, and preventing the progression of PD in PD mice.	107
Arterial injection	Lung ischemia-reperfusion injury (IRI) model	Gastrocnemius muscle of C57BL/6J male mice	Mouse lung tissues	*In vivo*	Isolated mitochondria were injected via pulmonary artery.	Twenty-four hours after reperfusion, mitochondrial transplantation improved lung mechanics and reduced lung tissue damage in mice.	19

### Mitochondria after transplantation

**Figure 3:**
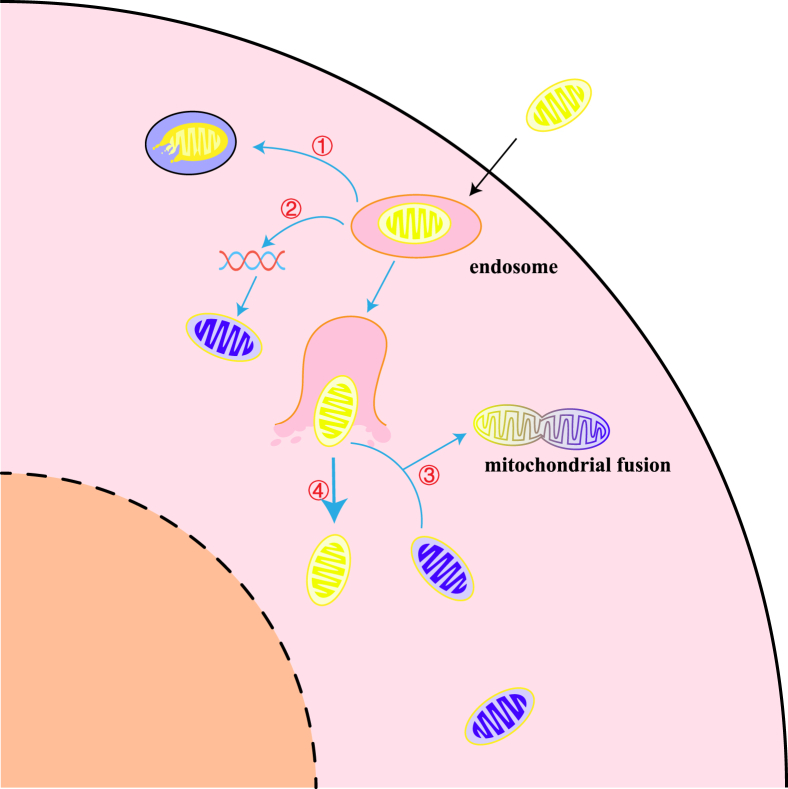
Possible endings of exogenous mitochondria after they enter the cell. Yellow mitochondria are exogenous mitochondria, and blue ones are recipient cell mitochondria. ① Endosomes bind to lysosomes, and mitochondria are degraded; ② Endosomes are in direct contact with the endogenous mitochondrial network and deliver mtDNA to endogenous mitochondria; ③ Endosomes rupture and exogenous mitochondria escape and fuse with endogenous mitochondria; ④ Endosomes rupture and exogenous mitochondria escape and do not integrate with the endogenous mitochondrial network.

Exogenous active mitochondria can be internalized into cells, where they may undergo one of two fates: binding to lysosomes and undergoing degradation or performing normal mitochondrial function and participating in cellular physiology (Fig. 3). However, the mechanism by which mitochondria evade endosomal surveillance after entering the cell remains inconclusive. It has been noted that exogenous mitochondria are internalized by cardiomyocytes and transferred to early endosomes within minutes, where the majority escape and fuse with the endogenous mitochondrial network while a small portion of mitochondria that do not escape are degraded after fusion with lysosomes in late endosomes.^109^ One theory suggests that, similar to viral escape endosomal surveillance, endosomes act as a kind of packaging for mitochondria and that there may be a mechanism for unpacking and leaking of cargo, which leads to the entry of mitochondria into the cytosol, but of course, this requires more relevant studies.^110^ Another theory is that direct contact between endosomes containing exogenous mitochondria and the endogenous mitochondrial network may deliver mitochondrial DNA to endogenous mitochondria, thereby rescuing cellular respiratory function.^110^

After evading endosomal surveillance, exogenous mitochondria can integrate into the endogenous mitochondrial network and perform their functions. It is well established that fusion and fission between mitochondria are constantly occurring to maintain mitochondrial homeostasis, a process known as mitochondrial dynamics, which can lead to a range of diseases once this mechanism is defective.^111^^,^^112^ Furthermore, mitochondria also rescue mitochondrial function in recipient cells by intercellular transfer via pathways such as TNTs and EVs.^85^^,^^113^ A study has shown that when mitochondria isolated from HeLa cells were transplanted into U2OS cells, 18 out of 22 recipient cells exhibited complete mitochondrial fusion, while 4 cells showed degradation of the transplanted mitochondria.^114^ This finding seems to imply that exogenous mitochondrial transplantation can be well fused into the mitochondrial network of the recipient cells and perform mitochondrial function. Another animal experiment demonstrated that brain infarct areas and neuronal death were attenuated in cerebrally ischemic rats after the transfer of allogeneic mitochondria; however, nearly no co-localized mitochondrial signals were detected in the neurons of the recipient rats, and it is clear that there is nearly no fusion or only a low percentage of fusion between internalized mitochondria and the host mitochondrial network.^115^ Despite the differing outcomes of these two studies, it can be concluded that exogenous mitochondrial transplantation is capable of restoring mitochondrial function in cells with existing mitochondrial dysfunction, at least for the duration of survival of the transplanted mitochondria.

## Mitochondrial transplantation therapy in lung diseases

**Figure 4:**
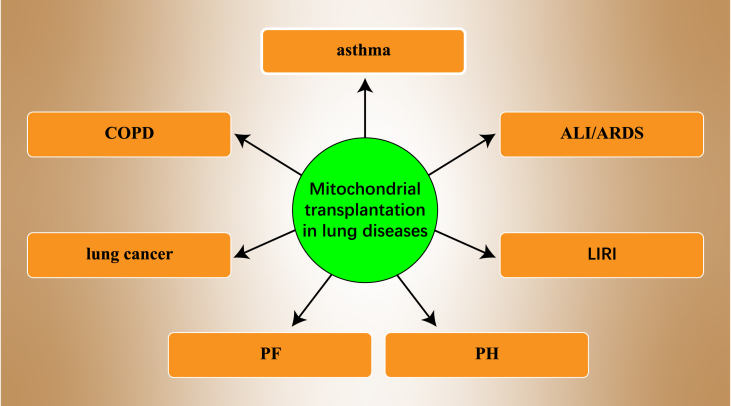
The applications of mitochondrial transplantation in lung diseases. COPD: chronic obstructive pulmonary disease; ALI/ARDS: acute lung injury/acute respiratory distress syndrome; LIRI: lung ischemia-reperfusion injury; PH: pulmonary hypertension; PF: pulmonary fibrosis.

Mitochondria play a crucial role in maintaining homeostasis in the lungs. However, due to various factors, such as the unique anatomical structure of the lungs, they are a frequent site of mitochondrial dysfunction. Over the past few decades, mitochondrial dysfunction has been observed in various types of lung cells, and these dysfunctional mitochondrial cells are closely associated with the development of lung diseases.^116^ This indicates that mitochondrial dysfunction plays a significant role in the pathogenesis and development of multiple lung diseases. Therefore, mitochondria may serve as potential therapeutic targets and promising avenues for the development of treatments for these lung diseases^30^ (Fig. 4). Mitochondrial transplantation therapy (MTT) is a promising therapeutic approach; however, while isolated studies have reported its application in pulmonary diseases, a comprehensive analysis remains lacking. Notably, MTT has demonstrated significant therapeutic efficacy in extra-pulmonary organs, including the brain,^117^ heart^118^ and liver.^119^ In contrast to these solid organs, pulmonary mitochondrial transplantation exhibits unique therapeutic characteristics. First of all, as discussed earlier, due to its special anatomical structure, the lungs are more prone to oxidative damage. Therefore, compared with that in other organs, mitochondrial dysfunction in lung diseases is more common, which also makes mitochondrial transplantation potentially more effective in lung diseases. Furthermore, beyond conventional vascular delivery, pulmonary mitochondrial transplantation can also be performed via mitochondrial aerosol atomization.^19^ Compared with vascular injection, this non-invasive mitochondrial transplantation method is more acceptable to patients. In addition, studies have pointed out that intravenously delivered mitochondria are widely distributed across multiple organs, such as the brain, liver, and kidney.^107^ This suggests that mitochondrial delivery via this method lacks tissue specificity and cannot achieve targeted delivery like that accomplished through the localized delivery of pulmonary aerosolization, resulting in lower delivery efficiency. Conversely, in mucus-obstructive lung diseases like COPD, viscous hyperconcentrated mucus may impede the access of exogenous mitochondria to lung cells via the airway.^120^ Given these distinctive considerations and the absence of a consolidated review, this comprehensive discussion was undertaken to evaluate the potential of mitochondrial transplantation for lung diseases.

### Chronic obstructive pulmonary disease (COPD)

COPD is a prevalent respiratory disorder characterized by persistent respiratory symptoms and airflow limitation. While the precise molecular mechanisms underlying the development of COPD remain incompletely understood, growing evidence implicates mitochondrial dysfunction as a central factor in its pathogenesis.^121^, ^122^, ^123^ A recent study has found several mitochondrial abnormalities in the lungs of COPD patients, including reduced mitochondrial size and activity, impaired mitochondrial fusion, enhanced mitochondrial autophagy, and lung cellular senescence, although the number of mitochondria was increased.^124^ As we have mentioned previously, damage to mitochondria induces the release of endogenous ROS from inflammatory cells (such as macrophages and neutrophils), leading to oxidative stress in the lungs (see Chapter 2.1). In turn, oxidative stress is a major driver of COPD and may promote COPD progression and increase complications through systemic oxidative stress.^125^ Therefore, given the importance in COPD, mitochondria may serve as a therapeutic target for the disease. In a cigarette smoke-induced rat model of COPD, mitochondria could be transferred from human-induced pluripotent stem cell-derived MSCs (iPSC-MSCs) to injured lung epithelial cells and attenuate the lung injury induced by cigarette smoke via co-incubation, and this transfer was associated with the formation of TNTs.^126^ In addition, another study has shown that mitochondrial transfer, which seems to be a stress adaptation mechanism, can occur between airway smooth muscle cells (ASMCs) in healthy ex-smokers at least partly via EVs, serving as a homeostatic mechanism to regulate mitochondrial and cellular functions in the airways and mitigate the consequences of mitochondrial dysfunction in COPD.^127^ These studies suggest that cells with mitochondrial dysfunction may spontaneously take up exogenous healthy mitochondria, regardless of whether they originate from homologous cells. In summary, these studies provide a mechanistic foundation for mitochondrial transplantation therapy in the treatment of COPD.

### Asthma

Consistent with COPD, the pathophysiology of asthma is also closely related to mitochondrial dysfunction and oxidative stress.^128^^,^^129^ An earlier study showed that mitochondrial dysfunction features such as mitochondrial ultrastructural changes (loss of cristae and swelling), decreased complex I expression, reduced ATP levels and cytochrome *c* oxidase activity were observed in bronchial epithelial cells of experimental allergic asthma mice and that IL-4 may be involved in the development of this dysfunction.^130^ For asthma treatment, some researchers have proposed that mitochondrial-targeted therapies, such as antioxidant treatment and mitochondrial replacement therapy, represent promising approaches. The transfer or reimplantation of healthy mitochondria into stressed cells can enhance the availability of functional mitochondria in recipient cells and supplement the relatively slow, multistage process of mitochondrial biogenesis.^131^ Yao et al observed mitochondrial transfer of iPSC-MSCs to airway epithelial cells with mitochondrial dysfunction via TNTs, which were formed by the Cx43 protein, and the results showed that local transplantation of iPSC-MSCs alleviated airway inflammation and protected the epithelial cells in asthma mice.^132^ Moreover, Ahmad et al showed that TNT-mediated mitochondrial transfer from MSCs to epithelial cells reversed allergic airway inflammation and attenuated airway hyperresponsiveness in asthmatic mice.^133^ These results show the beneficial effects of mitochondrial transfer on the treatment of asthma.

Interestingly, although asthma and COPD are pathophysiologically distinct diseases, both diseases are associated with airway remodeling and chronic inflammation and share some similar phenotypes, such as mitochondrial dysfunction and oxidative stress in airway epithelial cells. Therefore, therapies for asthma and COPD are somewhat similar, and it is not surprising that mitochondrial transplantation therapies work in a similar manner.

### Acute lung injury/acute respiratory distress syndrome (ALI/ARDS)

Acute lung injury (ALI) and its more severe manifestation, acute respiratory distress syndrome (ARDS), are life-threatening conditions resulting from excessive and uncontrolled systemic inflammatory responses to direct or indirect lung injury.^134^ A meta-analysis showed that elevated levels of mitochondrial dysfunction biomarkers (such as mtDNA, xanthine, hypoxanthine, glutathione, and malondialdehyde) are positively correlated with ARDS, with circulating mtDNA being the most commonly detected biomarker, suggesting that mitochondrial dysfunction plays a potential role in the pathogenesis of ARDS.^135^ Moreover, damaged mitochondria can release mitochondrial components (such as mtDNA/ox-mtDNA) through various pathways, which act as damage-associated molecular patterns (DAMPs) to activate inflammatory signaling pathways like NLRP3, TLR9/NF-κB, and cGAS-STING, thereby mediating the inflammatory response in the lungs of ALI/ARDS patients and exacerbating disease progression.^136^ Therefore, targeting mitochondria would be a promising therapy for ALI/ARDS. A study showed that bone marrow-derived mesenchymal stem cells (BMSCs) can restore mitochondrial dysfunction in alveolar epithelial cells and macrophages in lipopolysaccharide-induced ARDS mice, reverse the harmful changes in inflammatory factors such as IL-1β, TNF-α, and IL-10, and effectively improve ARDS symptoms in the mice.^137^ This may be mediated by the transfer of mitochondria from MSCs to damaged cells via MSC-derived microvesicles (MSC-MVs) and exosomes.^138^ One more direct evidence for mitochondrial transplantation comes from the study by Pang et al, who injected mitochondria isolated from rat soleus muscle into the jugular vein of ALI rats, demonstrating that mitochondrial transplantation could reduce endothelial damage to the alveolar–capillary barrier and improve gas exchange in the LPS-induced ALI experimental model.^139^

It has been reported that the global prevalence of ARDS accounts for 10% of ICU admissions, with a mortality rate of 35%–40%, making ARDS a significant public health issue that severely impacts human health.^140^ In addition to supportive therapies, a large number of studies using mitochondria as a therapeutic target for ALI/ARDS have been conducted. The number of their annual publications is rapidly increasing, among which studies on mitochondrial transfer have become a hot topic in recent years,^141^ suggesting that mitochondrial transplantation may become a feasible method for curing ALI/ARDS in future clinical practice.

### Lung ischemia-reperfusion injury (LIRI)

Lung ischemia-reperfusion injury (IRI) frequently occurs following lung transplantation and represents a leading cause of early postoperative mortality.^142^^,^^143^ Mitochondrial dysfunction is implicated in this pathological process. Indeed, studies have established ROS as a primary risk factor for IRI, with excessive ROS generation during ischemia-reperfusion (IR) triggering mitochondrial damage and cellular apoptosis.^144^^,^^145^ Animal models of LIRI have demonstrated that ischemia-reperfusion injury induces both mitochondrial dysfunction and elevated mtROS production in lung tissue, with the resultant mitochondrial impairment serving as a key mediator of pulmonary damage. Based on the above findings, some scholars believe that exogenous mitochondrial transplantation provides a unique therapeutic option for rescuing damaged cells in lung tissue.^146^ Research has shown that mitochondrial transplantation during *ex vivo* lung perfusion can alleviate tissue inflammation and ROS accumulation following lung IRI, improving lung function; therefore, mitochondrial transplantation therapy can be used during lung transplantation to attenuate IRI-induced graft rejection.^154^ Another study supports this view, indicating that mitochondrial transplantation significantly enhances lung mechanics and reduces lung tissue damage after ischemia-reperfusion, thereby decreasing morbidity and mortality in various clinical contexts, including lung transplantation, cardiopulmonary resuscitation, and pulmonary embolism.^19^

### Pulmonary hypertension (PH)

Pulmonary hypertension (PH) is a progressive disease primarily characterized by an abnormal increase in pulmonary artery pressure, which leads to pulmonary vascular obstruction, sclerosis, and vasoconstriction.^147^ The pathogenesis involves various cell types, including endothelial cells (ECs), smooth muscle cells (SMCs), and fibroblasts.^148^ Interestingly, these cells exhibit metabolic changes similar to those of cancer cells, which increase glycolytic metabolism through metabolic reprogramming, also known as the Warburg effect.^149^ This abnormal metabolism is fundamentally driven by mitochondrial dysfunction, which explains the observed mitochondrial dysfunction in PH, including defects in ETC, increased glycolysis, mtDNA damage, and imbalances in mitochondrial dynamics.^150^^,^^151^ For instance, a study by Plecitá-Hlavatá et al demonstrated that alterations in mitochondrial metabolism in fibroblasts drive significant changes in cellular behavior in the pathogenesis of PH vascular remodeling, further supporting the notion that mitochondrial dysfunction is a driving factor in PH.^152^ Here, mitochondrial transplantation has been proposed for the treatment of PH, and the feasibility of this therapy has been demonstrated. It was noted that mitochondria derived from femoral artery smooth muscle cells (FASMCs) were transplanted into the pulmonary arteries of rats with hypoxia-induced PH (HPH) by intravenous administration, and pulmonary vasoconstriction, pulmonary vascular remodeling, and pulmonary hypertension were effectively suppressed in these rats.^153^ A more recent study also found that exogenous mitochondrial transplantation increased ATP levels in lung tissue, reversed pulmonary vascular remodeling, and improved right ventricular function in PH rats.^154^

### Pulmonary fibrosis (PF)

Pulmonary fibrosis (PF) is a group of chronic, progressive lung diseases triggered by various factors that result in abnormal wound healing and lead to inflammation and excessive fibrosis, primarily affecting the lung interstitium.^155^ Idiopathic pulmonary fibrosis (IPF) is the most common form of PF, which seriously affects the quality of patient survival. There has been substantial evidence that mitochondrial dysfunction is a key factor driving the progression of PF. According to a report, alveolar epithelial cells (AECs), lung macrophages, and lung fibroblasts in PF exhibit mitochondrial dysfunction and increased ROS production, with AECs responding to ROS with apoptosis, and macrophages and fibroblasts exhibiting apoptosis resistance.^156^ In IPF, mitochondrial dysfunction includes changes in mitochondrial quantity, abnormalities in mitochondrial bioenergetics, alterations in mitochondrial dynamics, changes in mitophagy, and defects in mitochondrial DNA (mtDNA) damage and repair.^157^ A researcher found that the fluorescence intensity of lung tissues in IPF mice was significantly higher than that in normal mice when isolated fluorescence-tagged mitochondria were injected intravenously into bleomycin (BLM)-induced IPF mice, indicating that exogenous mitochondria were preferentially transported to and taken up by mitochondria-damaged cells and tissues.^158^ Huang et al demonstrated that human placental-derived MSCs (hMSCs) injected intravenously into BLM-induced PH mice resulted in mitochondrial transfer from hMSCs to damaged lung cells and that mice treated with mitochondrial transfer showed a significant reduction in lung fibrosis area and restoration of lung mitochondrial function compared to controls.^159^ These findings demonstrate the therapeutic potential of MTT and suggest that mitochondrial transplantation is a feasible and effective therapeutic option for PH treatment.

### Lung cancer

As is well known, cancer cells undergo metabolic reprogramming, shifting their metabolic pathways from oxidative phosphorylation to glycolysis, which is a hallmark of many malignant tumors. Currently, mitochondrial dysfunction has been observed in various types of cancer.^160^, ^161^, ^162^ These mitochondrial dysfunctions, caused by mutations in mtDNA, dysfunction of TCA cycle enzymes, electron leakage of ETC, and subsequent oxidative stress and/or aberrant oncogenic and tumor-suppressive signaling, alter the metabolic pathways of tumor cells, disrupt redox homeostasis and lead to resistance to apoptosis and treatment in tumor cells.^163^ A study has shown that breast cancer cells resistant to tamoxifen regained drug sensitivity and induced apoptosis and proliferation inhibition of resistant cells after mitochondrial dysfunction was reversed.^164^ However, another study found that increased mitochondrial dysfunction inhibited the proliferation and metastasis of non-small cell lung cancer (NSCLC) cells.^165^ A possible explanation for the similar outcomes resulting from the two opposite mitochondrial treatments is that severe mitochondrial dysfunction may lead to cell death and thus inhibit tumorigenesis, while mild mitochondrial dysfunction may enhance the generation of mtROS and redox rebalancing to stimulate cancer cell proliferation and invasiveness.^163^ Therefore, mild mitochondrial dysfunction may serve as a “comfort zone” for tumor cells, and exacerbating mitochondrial dysfunction and restoring mitochondrial function in tumor cells may serve as therapeutic strategies for cancer treatment. Mitochondrial transplantation falls into the latter category. Mitochondrial transplantation has a unique mechanism for inducing cancer cell death, including apoptosis (programmed cell death), necroptosis (non-programmed cell death), and parthanatos (a novel form of cell death), with the type of death depending on the cancer type.^166^ In malignant hepatocellular carcinoma, the transplantation of healthy mitochondria can delay the proliferation of liver cancer cells both *in vitro* and *in vivo* by blocking the cell cycle and inducing apoptosis.^57^ Furthermore, a study on breast cancer also showed that mitochondrial transplantation can induce breast cancer cell death by increasing the nuclear translocation of apoptosis-inducing factors.^21^ This study also pointed out that after transplantation of dysfunctional mitochondria, the original inhibition of cancer growth was eliminated, and the glycolytic metabolic phenotype increased.^21^ These results suggest that the anticancer effects of mitochondrial transplantation are not due to the toxic effects of exogenous mitochondria but rather through triggering the apoptotic mechanisms of tumor cells, leading to tumor cell death. Although a variety of tumor cells exhibit an inhibitory state after receiving healthy mitochondria, this phenomenon is controversial in lung cancer. Currently, radiotherapy is the primary treatment for NSCLC patients, and some scholars believe that mitochondria transported via extracellular vesicles into lung cancer cells may enhance the sensitivity to radiotherapy.^167^ However, a study by Wang et al showed that, after receiving mitochondria from lymphocytes, NSCLC cells exhibited enhanced cell proliferation, anti-cytotoxicity, and glucose metabolic reprogramming, suggesting that mitochondrial transfer promotes the malignant progression of tumors.^168^ Therefore, the effects of mitochondrial transplantation in lung cancer still require further research to be fully understood.

## Limitations and prospects

Mitochondrial transplantation therapy (MTT) has demonstrated good therapeutic efficacy in a variety of lung diseases, but there are still limiting factors that exist for mitochondrial transplantation. It has been found that isolated mitochondria can be stored on ice for approximately 1 h, beyond which the efficacy of the mitochondria is greatly reduced, and even after freezing the mitochondria and applying preservatives, the bioenergetic function of mitochondria decreases to less than 10%–15% of normal values.^169^ Therefore, how to maintain isolated mitochondrial activity will be a pressing issue in future work. Another factor limiting the application of mitochondrial transplantation is that it is still controversial whether this therapy causes rejection. There is no doubt that autologous mitochondrial transplantation does not cause inflammation and rejection, which has been demonstrated in animal experiments.^170^ However, in some cases, autologous mitochondrial transplantation is not suitable, such as in patients with congenital mitochondrial diseases. Therefore, allogeneic mitochondrial transplantation will be the focus of discussion. Ramirez-Barbieri et al showed that single or serial injections of allogeneic mitochondria did not elicit direct or indirect, acute or chronic alloreactivity, allorecognition or damage-associated molecular pattern molecule (DAMP) reaction.^171^ However, another study showed that allogeneic mitochondria induce the secretion of epithelial cells and adhesion molecules to initiate an inflammatory response.^172^ Therefore, more research support is still needed on whether allogeneic mitochondrial transplantation can induce rejection.

Regrettably, direct evidence regarding the efficacy of mitochondrial transplantation in aged lungs remains limited, and it cannot be directly proven that mitochondrial transplantation can exert beneficial effects on aging lungs. It is well established that mitochondrial dysfunction represents a hallmark of cellular senescence.^173^ Specifically, senescent cells exhibit reduced mitochondrial mass, diminished mitochondrial membrane potential, and accumulation of defective mitochondria.^63^ Some studies related to mitochondrial transplantation in aging cells/tissues may provide certain insights. Research has shown that healthy mitochondrial transplantation inhibits apoptosis, endoplasmic reticulum stress, and insulin resistance in aging MSCs and demonstrates improved anti-inflammatory activity in LPS-induced synovial cells.^174^ Another study also indicates that mitochondria isolated from the brains of young rats can effectively improve anxiety and depressive-like behaviors in aging rats subjected to chronic mild stress.^175^ These findings collectively demonstrate the rejuvenating potential of mitochondrial transplantation in aging contexts. Thus, synthesizing evidence across these models, it is plausible that transplantation of mitochondria derived from young donors may confer functional restoration in the senescent lung.

Overall, mitochondrial transplantation is a promising therapeutic modality for lung diseases, and although there are still some limitations, perhaps in the near future, mitochondrial transplantation will become a therapeutic option for a wide range of diseases (not only lung diseases) after these unknown factors have been overcome.

## CRediT authorship contribution statement

**Haoneng Wu:** Writing – original draft, Conceptualization. **Qiuran Zhao:** Visualization, Methodology. **Ying Zhao:** Validation, Methodology. **Jinguang Bai:** Validation, Methodology. **Junxi Pan:** Validation. **Songling Huang:** Writing – review & editing.

## Funding

This work was supported by Joint Special Fund of the Department of Science and Technology of Yunnan Province-Kunming Medical University, China (No. 202301AY070001-183) and Kunming Medical University Graduate Education Innovation Fund, China (No. 2024S245).

## Conflict of interests

The authors declare that they have no conflict of interests.
